# Growth performance, carcass traits and gut health of broiler chickens fed diets incorporated with single cell protein

**DOI:** 10.5713/ab.20.0844

**Published:** 2021-04-23

**Authors:** Gangavadi P. Hombegowda, Bypanahalli N. Suresh, Mysore C. Shivakumar, Puttamallappa Ravikumar, Bekkere C. Girish, Satturu M. Rudrappa, Huchamanadoddi C. Indresh

**Affiliations:** 1 Veterinary College, Karnataka Veterinary, Animal and Fisheries Sciences University, Hassan – 573 202, India; 2 Veterinary College, KVAFSU, Bangalore – 560 024, India

**Keywords:** Broilers, Carcass Quality, Growth Performance, Gut Health, Single Cell Protein

## Abstract

**Objective:**

This study was conducted to evaluate single cell protein (SCP), produced from *Methylococcus* species, as a protein source on the growth performance, carcass traits and gut health of broiler chickens.

**Methods:**

Ten iso-nitrogenous and iso-caloric diets containing 0 (Control), 2.5%, 5%, 7.5%, and 10% SCP replacing either soybean meal (T_1_ to T_5_) or fish meal (T_6_ to T_10_) were formulated. Each diet prepared for starter (0 to 14 days), grower (15 to 21 days), and finisher (22 to 42 days) phases was offered to four replicates of 10 chicks each (n = 400). Growth performance at different phases and carcass characteristics and intestinal morphology on 42nd day of trial were measured.

**Results:**

Body weight gain in groups fed 2.5% and 5% SCP diets were comparable to control during different phases and cumulatively, however lower (p<0.01) in 7.5% and 10% SCP diets. Feed conversion ratio was better (p<0.01) in 2.5% and 5% SCP diets. Dressing percentage, abdominal fat percentage and meat:bone ratio were not affected (p>0.05) by SCP inclusion in the diets. However, breast percentage was higher (p<0.01) in 2.5% and 5% SCP groups and thigh percentage higher in 7.5% and 10% SCP groups. Total microbial count in duodenum, jejunum and ileum were not affected (p>0.05) by SCP inclusion up to 10% in diets. Duodenal villi length and crypt depth were highest (p<0.01) in group fed 5% SCP diets and lowest in group fed 10% SCP diets. Jejunal villi length and crypt depth as well as ileal villi length were lowest (p<0.01) in group fed 10% SCP diets. Body weight gain, feed consumption, feed conversion ratio and gut health were better (p<0.01) in broilers fed fish meal based diets compared to soybean meal based diets.

**Conclusion:**

It was concluded that inclusion of SCP up to 5% replacing soybean meal in broiler diets is beneficial in improving growth rate, breast yield and gut health status.

## INTRODUCTION

Feed resource is a major constraint confronting the poultry industry as the feed alone accounts for 60 to 75 per cent of the cost of broiler chicken production. Among various constituents, protein fraction is one of the expensive components and constitute second most substantial nutrient in broiler diets. The fast-growing broiler chickens require high amount of protein in their diet with optimal amino-acid profiles. Protein and amino acids are essential for synthesis of various biologically active compounds such as enzymes, hormones and antibodies, each of which has a specific role in the body [1], besides requirement for maintenance and growth of body tissues [2].

Protein in modern poultry production practice is sourced from both vegetable (plant) and animal origin feed resources. Among protein meals from various oilseeds, soybean meal (SBM) is the most preferred source in broiler diets due to its high crude protein content (40% to 48%) with well-balanced amino acids coupled with high digestibility [3]. SBM meets approximately 80% of the protein and amino acid requirements of broiler birds at all stages [4]. However, use of SBM is sometimes limited because of poor quality due to presence of hulls and anti-nutritional factors such as trypsin inhibitor [5,6], due to fluctuation in its availability and variations in price with public concern about genetically modified plants [7]. Compared to SBM, other plant protein sources are generally imbalanced with essential amino acids and need to be supplemented with animal protein sources and crystalline amino acids to meet the requirements for critical amino acids for broiler production.

Fish meal, bone meal, meat meal, poultry meal and to a lesser extent blood meal and hydrolyzed feather meal are the important animal protein sources available for broiler diet formulations [8]. These sources offer a high level of protein, well balanced with essential amino acids, a high level of available phosphorus and reasonable amounts of other minerals [9]. However, their usage is limited due to variability in quality depending on the raw material and processing [10] and sometimes presence of pathogenic microorganisms. Fish meal is expensive and its inclusion in broiler diet is also restricted due to development of fishy taint in meat and phosphorus imbalance. Apart from ensuing protein or amino acids supply to broiler chickens, choosing ingredients to maximize nutrient availability, promoting gut health and immunity is necessary [11,12]. Such protein products are highly digestible, devoid of anti-nutritional factors and contains biologically active components. These functional ingredients inclusion in broiler diets, especially at an earlier age of birds, can assist early gut development and digestive physiology, and improve broilers’ growth performance [13].

In view of above, there is a constant search for high-quality protein feed sources which can improve broiler chicken production. Single cell proteins (SCP) are such unique ingredients to supply protein and amino acids in poultry diets. SCPs are broad range of very diverse products produced from microorganisms as fungi, algae (*Spirulina, Chlorella*), yeasts (*Saccharomyces cerevisiae*) or bacteria (*Methylococuus, Cyanobacteria*) The high growth rates of micro-organisms, their high efficiency of substrate conversion and protein synthesis forms the basis for the production of SCP [14]. The possibility of large-scale production from high-tech bioreactors using inexpensive raw materials and a continuous harvest are other advantages of SCP [14–16]. SCPs contains ~65% crude protein and has an amino acid profile similar to fishmeal [17]. Also, SCP is produced by a fermentation process and hence can enable traceability and consistency. This is a huge advantage compared to existing sources of protein and also safety and no contamination can be ensured in SCP products. Studies indicated that SCPs can be included up to 5% level or to meet 30% to 40% of total crude protein in broiler diets with improved weight gain, feed conversion ratio (FCR) and economic efficiency in broilers [18–21]. In pigs, SCP replacing 50% of fish meal in their diet had similar growth performance, nutrient digestibility and intestinal morphology [22]. However, due to large variation in composition and characteristics of individual SCPs, suitable inclusion levels should always be determined per source of biomass [23]. Further, more information needs to be established on the gut health promoting activities of SCP in broiler chickens. Hence, a study was conducted to assess the effect of incorporation of SCP at graded levels on the performance, carcass traits, serum biochemistry and gut health of broiler chickens.

## MATERIALS AND METHODS

The protocols used in the experiment was approved by the Institutional Animal Ethics Committee of Veterinary College, Hassan, India as per the guidelines of the Committee for the Purpose of Control and Supervision of Experimentation on Animals (CPCSEA), Government of India, New Delhi.

### Source and analysis of single cell proteins

The SCP (StringPro, rebranded as PRO-dg) used in the trial was supplied by M/s String Bio Pvt Ltd., Bangalore, India. The organisms employed to produce SCP were methanotrophs, the primary strain is a *Methylococcus*. This organism is a BSL1 organism that has European Commission approval for use in feed. The SCP was produced through a fermentation process using methane as a carbon source and is a more sustainable, traceable and consistent product. The SCP sample procured in single batch was analysed for proximate principles namely dry matter, crude protein, ether extract and crude fiber as per the methods described by AOAC [24] while the nitrogen free extract was calculated as the difference. The amino acid profile of SCP and other feed ingredients were analysed using high performance chromatography (Agilent 1260 Infinity II; Agilent Technologies, Santa Clara, CA, USA) for formulation of experimental diets and the values are presented in Table 1.

### Experimental diets

Five iso-nitrogenous and iso-caloric diets were prepared by incorporating SCP at 0%, 2.5%, 5%, 7.5%, and 10% levels replacing SBM to meet the nutrient requirements specified by Indian Council of Agricultural Research (ICAR, 2013) [25]. Similarly, second set of five diets were prepared by incorporating SCP at 0%, 2.5%, 5%, 7.5%, and 10% levels replacing fish meal. The diets in mash form were prepared separately for each phase i.e., starter (0 to 14 days), grower (15 to 21 days), and finisher (22 to 42 days) phases. The ingredient and nutrient composition of starter, grower and finisher diets are presented in Table 2, 4, and 5, respectively.

### Experimental birds

A total of 400 one-day-old straight run commercial chicks (Vencobb strain) were wing banded and randomly divided into 40 replicates of 10 chicks each. Each of the 10 diets were offered *ad libitum* to four replicate groups of 10 chicks each in completely randomised design. The birds were kept in deep litter system under well-ventilated house. The brooding was done up to 2 weeks of age as per the standard practice and all the birds were vaccinated against New Castle disease on 7th and 21st day and against Infectious Bursal disease on 14th and 28th day of age. The rest of the managemental practices were applied uniformly to all the birds during the 42-day experimental period.

### Growth parameters

All the birds were individually weighed and replicate wise feed intake were recorded at weekly intervals. The feed conversion ratio was calculated on the basis of unit feed intake to unit gain in body weight for each replicate. The mortality of birds was recorded as and when occurred and subjected for post mortem examination to ascertain the cause.

### Carcass evaluation

At the terminal day of experiment (42nd day), two birds (one male and one female) from each replicate (8 per treatment) were randomly selected, starved for 12 hours with the provision of *ad libitum* water and then weighed and sacrificed as per Modified Kosher method [26] for carcass evaluation. The data in terms of weight of dressed chicken, breast meat, wings, thigh, drum stick, abdominal fat and various visceral and lymphoid organs viz., liver, gizzard, heart, spleen, thymus and bursa (g/100 g pre-slaughter body weight) were recorded. The meat to bone ratio was calculated by dividing weight of thigh meat by weight of thigh bone.

### Microbial evaluation and pH of intestinal contents

The entire intestine from each slaughtered broiler chicken was aseptically removed and for each section of the intestine, one end was cut and the contents around 1.0 g was squeezed into 0.1% peptone water solution. The dilutions were prepared with the same diluent and aliquots of 10 μL and 100 μL were plated on selective nutrient culture media. Then plates were incubated anaerobically at 37°C for 48 to 72 hours. The total number of surface viable bacterial cell counts were carried out by standard plate count technique in nutrient agar plates and expressed as number of colony forming units per gram (cfu/g) of intestinal content. pH of duodenal content was recorded using portable pH meter (pHep; Hanna Instrument, Woonsocket, RI, USA) at the time of slaughtering of birds.

### Intestinal morphometry

The section of duodenum, jejunum and ileum segments of small intestine was taken out from the slaughtered birds and each sample was transferred to containers containing 10% neutral buffered formalin for fixation. After 12 to 24 hrs of fixation period, samples were washed overnight in running tap water, then they were transferred to alcohol for dehydration and then to xylene for clearing and then embedded in paraffin, sectioned to a 2 to 5 μm thickness, mounted on glass slides and stained with hematoxylin-eosin. The villi height and crypt depth were then measured by using a trinocular stereoscopic microscope (Lynx; LynxBio, San Diego, CA, USA) under 4× magnification. The images were captured by a camera coupled to the microscope and connected to an image analyzer (T-Capture; Tucsen Photonics Co. Ltd., Fuzhou, Fujian, China) and measured using the paint brush software. A minimum of five villi and crypt were scored for each bird, then means calculated were used in the statistical analysis.

### Statistical analysis

The data obtained during the trial was subjected for two way analysis of variance for the treatments and the main effect of dietary regime (SCP level and protein source). All the statistical procedures were carried out as per the procedures of Snedecor and Cochran [27]. Duncan’s multiple range test [28] was used to compare the means.

## RESULTS AND DISCUSSION

### Chemical composition of single cell proteins

The proximate analysis of SCP revealed that dry matter, organic matter, crude protein, ether extract, crude fiber, and nitrogen free extract were 95.34%, 90.38%, 61.78%, 7.54%, 2.33%, and 18.73%, respectively on as such basis and the values are similar to those reported by An et al [28]. The amino acid profile of SCP (Table 1) was very much comparable to that of the amino acid profile of fish meal. The SCP carries high amount of total essential amino acids compared to fishmeal. However, sulfur containing amino acids (methionine and cysteine) were marginally lower in SCP when compared to fish meal. The amino acid profile of SCP sample was similar to the values reported by White and Balloun [29]. Further, Srividya et al [30] reported that the yield and chemical composition of SCP microorganisms may vary with the type of substrate used, culture conditions maintained and specific organism used during the production process.

### Growth performance

The mean body weight gains (g/bird) of broiler birds among different treatments were different (p<0.01) during all phases and cumulatively (Table 5). Pertaining to SCP levels, the groups fed 2.5% or 5% SCP diets showed higher (p<0.01) body weight gain when compared to control group during different phases and cumulatively. Further, phase wise and cumulative mean body weight gains were higher (p<0.01) in fish meal based diets compared to SBM based diets. Waldroup and Payne [31], Najib [32] and Pourelmi et al [33] reported that inclusion of 10% and 15% bacterial cell protein replacing SBM in broiler diets resulted in significantly decreased body weight gain compared to control and 5% bacterial cell protein diet groups. Skrede et al [17] also observed reduced growth rates of broiler birds at 8% and 10% SCP inclusion levels in their diets replacing SBM. The reduced body weight gain was attributed to reduced feed intake associated with the powdery nature of the bacterial or yeast protein at levels greater than 15 percent [31]. Similarly, in the present study, finer SCP particles resulted in clump formation around the beak of the birds fed diets containing 7.5% and 10% SCP which resulted in decreased total feed intake and body weight gain of broilers at higher level of SCP inclusion. The higher body weight gain in fish meal based diets is due to higher bio-availability of protein and amino acids in fish meal than SBM [34].

The mean feed intake of boiler birds among different treatments were different (p<0.01) during different phases and cumulatively (Table 5). The SCP levels (0%, 2.5%, 5%, 7.5%, and 10%) and protein source (SBM and fish meal) ensued difference (p<0.01) in feed intake through different phases and cumulatively. Waldroup and Payne [31] also reported that the reduction in feed intake is associated with the powdery nature of the bacterial protein. Such changes in feeding behaviour was also reported by White and Balloun [35] at 9% and 12% SCP inclusion in diets. Schoyen et al [36] reported that 6% bacterial protein meal in the diet replacing SBM or fishmeal reduced the feed intake. An et al [28] indicated that *Corynebacterium ammoniagenes* derived SCP at 5% in broiler diet replacing SBM resulted in decreased feed intake which was associated with free adenine or unknown components present in bacteria-derived SCP. Overland et al [37] indicated that reduced feed intake was due the decreased palatability with increasing bacterial protein autolysate associated with the greater proportions of free amino acids and resulted in decreased performance of birds. In this study there were no negative effects observed at higher percentage of inclusion suggesting that the powdery form of the protein has lowered the feed intake. Thus, optimization of the particle size is required to include the SCP at higher percentages in the diet.

The higher feed intake in fish meal based diet than SBM based diet is attributed to the fact that fish meal improves the palatability of the feed and increases feed intake [34]. The usefulness of a protein feedstuff for poultry depends upon its ability to supply a sufficient amount of the essential amino acids that the bird requires, as well as the protein digestibility and the level of toxic substances associated with it [38].

The mean FCR (kg feed consumed to kg live weight gain) among treatments were different (p<0.01) different during all phases and cumulatively (Table 5). In general, better FCR at 5% SCP inclusion compared to control was observed. Waldroup and Payne [31] and Pourelmi et al [33] observed that the incorporation of SCP at 10% and 15% in broiler diet replacing SBM resulted in lowered (p<0.01) FCR compared with control. Contrarily, Overland et al [37] reported improved FCR (p<0.05) due to inclusion of bacterial protein autolysate at 12% replacing SBM in broiler diet. In our study, improved FCR was seen at 2.5% and 5% inclusion levels of SCP in the diet.

The percent of livability of birds were similar (p>0.05) among different treatments. As per main component SCP levels, the incorporation of SCP resulted in increased (p< 0.05) livability compared to control under all inclusion levels (Table 3). However, Pourelmi et al [33] reported that chickens fed diet containing 10% and 15% SCP had 40% and 100% mortality respectively. The livability values were non-significant (p>0.05) between SBM and fish meal based diets indicating that the source of protein in the diets has no influence on livability of broiler birds.

### Carcass traits

The dressing percentage values (Table 6) were non-significant (p>0.05) among various treatments indicating that incorporation of SCP up to 10% in the diet has no influence on dressed carcass weight. The same was also confirmed by Najib [32] who observed that inclusion of SCP at 5%, 10%, and 15% by replacing SBM in the broiler ration has no significant effect on dressing percentage. Whereas, Overland et al [37] observed that inclusion of bacterial protein autolysate at 8% and 12% of the diet replacing SBM in the broiler diet showed significantly lower carcass weight and dressing percentage compared to the control diet group. It was opined that the variation in the result of dressing percentage might be due to use of different strain of the yeast or SCP used or manipulation of some other ingredients in the starter ration [20].

The breast percentage of broilers among various treatments were found highly significant (p<0.01) and with respect the main component SCP levels, significantly (p<0.01) higher breast percentage values were observed in broilers fed 2.5% and 5% SCP incorporated diets (Table 4). The results were similar with the findings of Yadav et al [39] who observed that supplementation of probiotic (*Bacillus subtilis*) at the level of 1 mg/g of diet has significant (p<0.01) effect on weight of the breast muscle. Nahanshon et al [40] opined that adding bacterial probiotic to diet enhances the protein availability and the carcass weight increases with increasing the protein content of diet. In contrary, An et al [28] noticed that incorporation of graded levels of *Corynebacterium ammoniagenes* derived SCP up to 5% (50 g/kg) of ration by replacing SBM in the broiler diet did not affected the relative weight of breast muscle significantly (p>0.05). Further, the main component protein source showed non-significant (p>0.05) between SBM and fish meal groups.

The thigh percentage of live weight among various treatments and SCP levels as main component were found highly significant (p<0.01). The broilers fed with 7.5% and 10% SCP incorporated diets showed significantly (p<0.01) increased thigh percentage. The increased thigh percentage is due to their ability to enhance synthesis and bioavailability of nutrients along with positive effects on intestine activity and increasing digestive enzymes and thereby promoting growth of muscle tissues [41]. In contrary, Schoyen et al [42] observed replacing SBM and fish meal with graded level of bacterial protein meal (2%, 4%, and 6%) has no significant on weight of thigh.

The drumsticks percentage of live weight among various treatments and main components (SCP level and protein source) were non-significant (p>0.05) across all diets. The abdominal fat percentage of live weight were found comparable (p>0.05) among various treatments and main components (SCP level and protein source) which is similar to findings of Overland et al [37]. The meat to bone ratio were highly significant (p<0.01) among various treatments and protein sources (SBM and fish meal diets) while no such differences were observed when statistically analysed as per main component SCP levels.

### Organometry

The relative weight of giblet organs viz., liver, heart and gizzard and lymphoid organs viz., bursa of Fabricious, spleen and thymus were significantly (p<0.01) different among different treatments and SCP levels as a main component (Table 6). Similarly, Najib [32] observed significantly (p<0.05) different liver weight in broiler birds fed 5%, 10%, and 15% SCP based diets. However, Inam et al [44] noticed no significant difference in gizzard, heart and liver weight in broiler chicken fed diets containing 5%, 10%, and 15% *Saccharomyces cerevisiae* replacing SBM. An et al [28] observed that incorporation of graded levels of *Corynebacterium ammoniagenes* derived SCP up to 5% of broiler diet did not affected the relative weights of spleen and bursa of Fabricious. Pourelmi et al [33] also observed no effect with respect to the absolute weights of the liver, gizzard, spleen and bursa of Fabricious due to SCP inclusion at 5%, 10%, and 15% in broiler diets replacing SBM. Further, non-significant (p>0.05) difference in giblet and lymphoid organs weight was noticed for main component protein source.

### Microbial count and pH of intestinal content

The microbial count of intestinal content from duodenum, jejunum, ileum and ileo-caecal junction were similar (p>0.05) among the treatments, SCP levels and between protein source (Table 7) The results are in accordance with Pourelmi et al [33] who observed that incorporation of SCP up to 10% of the broiler diet by replacing SBM resulted in non-significant (p>0.05) difference on gut microbial population.

The pH of intestinal content among the treatments and between protein source were non-significant (p>0.05). However, the pH of intestinal content showed significant (p<0.05) difference for SCP levels as a main component. Hassanein and Soliman [44] observed that supplementation of *Saccharomyces cerevisiae* in basal diet of laying hens at 0.4%, 0.8%, 1.2%, and 1.6% showed non-significant effect on ileal pH.

### Intestinal tract morphometry

The duodenal villi length was higher (p<0.01) in groups fed 5% SCP diets and lower in groups fed 7.5% and 10% SCP diets compared to control (Table 8). The crypt depth in various groups was comparable (p>0.05) among SBM based diets but the crypt depth was higher (p<0.01) in groups fed 5% SCP diets compared to control in fish meal diets. The duodenal villi/crypt depth ratio was comparable among SBM based and fish meal diets fed groups. Analysis of the effect of main component SCP levels i.e., 0%, 2.5%, 5%, 7.5%, and 10% SCP on duodenal villi length, crypt depth and villi/crypt ratio revealed variations (p<0.01 or p<0.05) among the treatment groups. The villi length, crypt depth and the villi/crypt depth ratio were higher (p<0.01) in 5% SCP diet fed group compared to control, 2.5%, 7.5%, and 10% SCP diet fed groups. In main component protein sources, duodenal villi length, crypt depth and villi/crypt ratio showed non-significant (p>0.05) variation between SBM and fish meal groups. The incorporation of SCP at 5% in diets showed beneficial effect on the gut health of duodenum of broiler birds.

Among different treatments, jejunal villi length and crypt depth were lower (p<0.01) in 10% SCP diet fed group compared to control, 2.5%, 5%, and 7.5% SCP diets fed groups. Analysis of the effect of SCP levels revealed higher (p<0.01) jejunal villi length and the crypt depth in 5% SCP diet fed group but lower (p<0.01) in 10% SCP diet fed group compared to control, 2.5%, 5%, and 7.5% SCP diets fed groups. Analysis of the main component protein sources revealed higher (p<0.05) jejunal villi length and crypt depth in SBM diets compared to fish meal diets. However, jejunal villi/crypt ratio were comparable (p>0.05) among different treatments, SCP levels and protein source. The incorporation of SCP at 5% in diets had beneficial effect on jejunal morphometry while SCP at 10% reduced villi length and crypt depth in duodenum of broiler birds.

The ileal villi length, crypt depth and villi/crypt depth ratio revealed non-significant (p>0.05) variations among the treatment groups, SCP levels and protein source except ileal villi length which was significantly (p<0.05) lower in 10% SCP incorporated diet fed group compared to 0%, 2.5%, 5%, and 7.5% SCP incorporated diet fed groups. The results indicated that inclusion of SCP at 2.5%, 5%, and 7.5% had no adverse effect on ileal health while inclusion of SCP at 10% significantly reduced the villi length and crypt depth.

In the present study, the villi/crypt depth ratio in the duodenum, jejunum and ileum revealed non-significant variations among the treatment groups. The main component protein source also had no significant effect on the villi/crypt depth ratio in various segments of small intestine. These results are in accordance with the Inam et al [43] who reported that inclusion of *Saccharomyces cerevisiae* in the broiler starter ration by replacing SBM at the rate of 5%, 10%, and 15% showed no significant (p>0.05) effect on intestinal morphology like villus height and crypt depth. Samanya and Yamauchi [45] also reported that supplementation of *Bacillus subtilis* at 0%, 0.2%, 0.5%, and 1% levels to the basal mash diet up to 28 days showed significantly (p<0.05) higher villus length of the duodenum in the 0.2% group compared to the control group. Latorre et al [46] recorded significant reduction in villi length:crypt depth ratio and he attributed the same to higher presence of insoluble fibre in the intestinal digesta, therefore increasing cellular turnover. The improvement in villi length in the duodenum, jejunum and ileum upon incorporation of SCP could be due to an elevated production of short chain fatty acids by lactic acid bacteria in the intestinal lumen and also production of antimicrobial and antioxidant compounds with a reduction of substrates available in the intestinal lumen which could diminish the bacterial population of Gram-negative and anaerobic bacteria, therefore decreasing the level of intestinal inflammation and enhancing epithelial integrity, nutrient absorption and bone strength and composition.

## CONCLUSION

Single cell protein produced from natural gas offers a more sustainable, high-quality, safe and traceable alternative to conventional protein sources used in poultry feed. The study showed that SCP can be included up to 5% in the commercial broiler diets as a protein source replacing SBM or fish meal without any adverse effect on the growth performance of broiler birds besides improving gut health status. The inclusion of SCP upto 5% results in a better FCR compared to traditional protein sources.

## Figures and Tables

**Table 1:** Amino acid content of different protein sources

Amino acid (g/kg)	Single cell protein	Fish meal	Soybean meal
Lysine	49.2	50.5	28.2
Methionine	7.3	19.7	61
Cysteine	4.5	6.4	6.47
Threonine	32.5	31.8	18.0
Tryptophan	18.5	6.8	6.2
Isoleucine	32.6	29.5	21.1
Leucine	58.1	42.8	35.6
Histidine	22.8	15.9	12.5
Phenylalanine	42.5	25.0	23.9
Tyrosine	27.6	20.0	11.9
Valine	39.8	33.6	22.0
Aspartic acid	64.1	60.0	53.6
Serine	25.8	27.6	23.5
Glutamic acid	74.1	87.1	82.5
Proline	26.2	30.6	23.2
Glycine	39.1	48.2	19.8
Alanine	61.7	41.7	20.1
Arginine	39.9	40.1	34.2
Total	666.3	617.4	448.5

**Table 2:** Ingredient and nutrient composition of experimental broiler diets compounded for starter phase (1 to 14 days)

Items	Starter phase (1 to 14 d)									

	T_1_	T_2_	T_3_	T_4_	T_5_	T_6_	T_7_	T_8_	T_9_	T_10_
Ingredient (kg)										
Maize	530.4	546.9	563.3	580.1	596.8	640.7	628.9	617.9	607.8	596.8
Fish meal-50	0	0	0	0	0	100.0	75.0	50.0	25.0	0
Soybean meal-45	385.5	343.6	301.8	259.5	217.0	223.5	223	221.2	218.5	217.0
Di-calcium phosphate	18.8	19.2	19.6	20	20.4	3.6	7.8	12.0	16.2	20.4
Lime stone powder	14.4	14.1	13.74	13.4	13.1	10.7	11.3	11.9	12.5	13.1
Salt	4.5	4.5	4.5	4.5	4.6	1.1	2.0	3.0	3.7	4.6
Trace minerals^1)^	1.0	1.0	1.0	1.0	1.0	1.0	1.0	1.0	1.0	1.0
Soya oil	42.0	41.5	40.9	40.3	39.6	17.1	23	28.7	34	39.6
L-lysine	0.2	0.59	0.96	1.3	1.8	0	0	0.6	1.3	1.8
DL-methionine	2.4	2.85	3.35	3.9	4.4	1.5	2.2	2.9	3.7	4.4
L-threonine	0	0	0.1	0.2	0.5	0	0	0	0.5	0.5
Vitamin premix^2)^	0.25	0.25	0.25	0.25	0.25	0.25	0.25	0.25	0.25	0.25
Single cell protein	00	25.0	50.0	75.0	100.0	00	25.0	50.0	75.0	100.0
Additives	0.8	0.8	0.8	0.8	0.8	0.8	0.8	0.8	0.8	0.8
Total	1,000	1,000	1,000	1,000	1,000	1,000	1,000	1,000	1,000	1,000
Crude protein (%)	22.00	22.00	22.00	22.00	22.00	22.00	22.00	22.00	22.00	22.00
ME (kcal/kg)	3,000	3,000	3,000	3,000	3,000	3,000	3,000	3,000	3,000	3,000
Calcium (%)	1.0	1.0	1.0	1.0	1.0	1.0	1.0	1.0	1.0	1.0
Av phosphorous (%)	0.45	0.45	0.45	0.45	0.45	0.45	0.45	0.45	0.45	0.45
Lysine (%)	1.20	1.20	1.20	1.20	1.20	1.24	1.20	1.20	1.21	1.20
Meth+Cyst (%)	0.86	0.86	0.86	0.86	0.86	0.86	0.86	0.86	0.86	0.86
Threonine (%)	0.82	0.80	0.80	0.80	0.81	0.83	0.82	0.80	0.83	0.81
Linoleic acid (%)	1.30	1.32	1.33	1.34	1.36	1.65	1.58	1.50	1.43	1.36

ME, metabolizable energy.

1) Trace minerals contains Fe, 38 g; Mn, 35 g; Cu, 7 g; Zn, 35 g; I, 1.25 g; Cr, 0.150 g; Se, 0.168 g.

2) Vitamin premix contains Vit A, 22.50 MIU; Vit D_3_, 4.50 MIU; Vit E, 60.0 g; Vit K, 8.0 g; Vit B_1_, 4.0 g; Vit B_2_, 20.0 g; Vit B_6_, 6.0 g; Vit B_12_, 0.03 g; Niacin, 60.0 g; Calcium-D-pantothenate, 30.0 g; Folic acid, 4.0 g; Biotin, 0.20 g; Vit C, 100.00 g.

**Table 3:** Ingredient and nutrient composition of experimental broiler diets compounded for grower phase (15 to 21 days)

Items	Grower phase (15 to 21 d)									

	T_1_	T_2_	T_3_	T_4_	T_5_	T_6_	T_7_	T_8_	T_9_	T_10_
Ingredient (kg)										
Maize	538.5	553.7	570.3	585.2	600.9	650.1	633.8	624.7	612.6	600.9
Fish meal-50	0	0	0	0	0	100.0	75.0	50.0	25.0	0
Soybean meal-45	375.4	335.0	293.3	253.0	211.5	212.1	212.0	212.4	212.5	211.5
Di-calcium phosphate	16.0	16.4	16.7	17.1	17.5	0.8	5.0	9.1	13.3	17.5
Lime stone powder	14.9	14.5	14.3	13.9	13.6	11.2	11.8	12.5	13.0	13.6
Salt	4.5	4.5	4.5	4.5	4.6	1.1	2.0	3.0	3.7	4.6
Trace minerals^1)^	1.0	1.0	1.0	1.0	1.0	1.0	1.0	1.0	1.0	1.0
Soya oil	47.6	47.3	46.8	46.6	46.3	22.4	28.4	34.6	40.4	46.3
L-lysine	0	0	0	0	0.2	0	0	0	0	0.2
DL-methionine	1.3	1.8	2.3	2.8	3.4	0.5	1.2	1.9	2.6	3.4
L-threonine	0	0	0	0.1	0.2	0	0	0	0.1	0.2
Vitamin premix^2)^	0.25	0.25	0.25	0.25	0.25	0.25	0.25	0.25	0.25	0.25
Single cell protein	00	25.0	50.0	75.0	100.0	00	25.0	50.0	75.0	100.0
Additives	0.8	0.8	0.8	0.8	0.8	0.8	0.8	0.8	0.8	0.8
Total	1,000	1,000	1,000	1,000	1,000	1,000	1,000	1,000	1,000	1,000
Crude protein (%)	21.50	21.50	21.50	21.50	21.50	21.50	21.50	21.50	21.50	21.50
ME (kcal/kg)	3,050	3,050	3,050	3,050	3,050	3,050	3,050	3,050	3,050	3,050
Calcium (%)	0.95	0.95	0.95	0.95	0.95	0.95	0.95	0.95	0.95	0.95
Av phosphorous (%)	0.40	0.40	0.40	0.40	0.40	0.40	0.40	0.40	0.40	0.40
Lysine (%)	1.16	1.13	1.11	1.08	1.07	1.21	1.17	1.13	1.09	1.07
Meth+Cyst (%)	0.76	0.76	0.76	0.76	0.76	0.76	0.76	0.76	0.76	0.76
Threonine (%)	0.80	0.79	0.78	0.78	0.78	0.82	0.80	0.79	0.78	0.78
Linoleic acid (%)	1.31	1.33	1.34	1.35	1.36	1.67	1.59	1.51	1.44	1.36

ME, metabolizable energy.

1) Trace minerals contains Fe, 38 g; Mn, 35 g; Cu, 7 g; Zn, 35 g; I, 1.25 g; Cr, 0.150 g; Se, 0.168 g.

2) Vitamin premix contains Vit A, 22.50 MIU; Vit D3, 4.50 MIU; Vit E, 60.0 g; Vit K, 8.0 g; Vit B_1_, 4.0 g; Vit B_2_, 20.0 g; Vit B_6_, 6.0 g; Vit B_12_, 0.03 g; Niacin, 60.0 g; Calcium-D-pantothenate, 30.0 g; Folic acid, 4.0 g; Biotin, 0.20 g; Vit C, 100.00 g.

**Table 4:** Ingredient and nutrient composition of experimental broiler diets compounded for finisher phase (22 to 42 days)

Items	Finisher phase (22 to 42 d)									

	T_1_	T_2_	T_3_	T_4_	T_5_	T_6_	T_7_	T_8_	T_9_	T_10_
Ingredient (kg)										
Maize	603.3	619.0	634.5	650.0	666.3	714.1	702.1	690.3	678.6	666.3
Fish meal-50	0	0	0	0	0	100.0	75.0	50.0	25.0	0
Soybean meal-45	319.0	278.2	237.4	1964	154.7	156.2	156.1	155.6	154.9	154.7
Di-calcium phosphate	15.1	15.5	15.9	16.3	16.6	00	4.1	8.30	12.5	16.6
Lime stone powder	12.9	12.5	12.2	12.0	11.7	9.1	9.8	10.3	10.9	11.7
Salt	3.2	3.2	3.2	3.2	3.3	0	0.7	1.6	2.4	3.3
Trace minerals^1)^	1.0	1.0	1.0	1.0	1.0	1.0	1.0	1.0	1.0	1.0
Soya oil	43.5	43.1	42.8	42.6	42.1	18.5	24.4	30.3	36.2	42.1
L-lysine	0	0	0	0	0.3	0	0	0	0	0.3
DL-methionine	1.2	1.7	2.2	2.7	3.2	0.3	1.0	1.8	2.7	3.2
L-threonine	0	0	0	0	0	0	0	0	0	0
Vitamin premix^2)^	0.25	0.25	0.25	0.25	0.25	0.25	0.25	0.25	0.25	0.25
Single cell protein	00	25.0	50.0	75.0	100.0	00	25.0	50.0	75.0	100.0
Additives	0.8	0.8	0.8	0.8	0.8	0.8	0.8	0.8	0.8	0.8
Total	1,000	1,000	1,000	1,000	1,000	1,000	1,000	1,000	1,000	1,000
Crude protein (%)	19.50	19.50	19.50	19.50	19.50	19.50	19.50	19.50	19.50	19.50
ME (kcal/kg)	3,100	3,100	3,100	3,100	3,100	3,100	3,100	3,100	3,100	3,100
Calcium (%)	0.80	0.80	0.80	0.80	0.80	0.80	0.80	0.80	0.80	0.80
Av phosphorous (%)	0.38	0.38	0.38	0.38	0.38	0.38	0.38	0.38	0.38	0.38
Lysine (%)	1.02	0.99	0.97	0.94	0.94	1.07	1.03	0.99	0.95	0.94
Meth+Cyst (%)	0.70	0.70	0.70	0.70	0.70	0.70	0.70	0.70	0.70	0.70
Threonine (%)	0.73	0.71	0.70	0.69	0.68	0.74	0.73	0.71	0.69	0.68
Linoleic acid (%)	1.42	1.43	1.45	1.46	1.47	1.77	1.70	1.62	1.55	1.47

ME, metabolizable energy.

1) Trace minerals contains Fe, 38 g; Mn, 35 g; Cu, 7 g; Zn, 35 g; I, 1.25 g; Cr, 0.150 g; Se, 0.168 g.

2) Vitamin premix contains Vit A, 22.50 MIU; Vit D_3_, 4.50 MIU; Vit E, 60.0 g; Vit K, 8.0 g Vit B_1_, 4.0 g; Vit B_2_, 20.0 g; Vit B_6_, 6.0 g; Vit B_12_, 0.03 g; Niacin, 60.0 g; Calcium-D-pantothenate, 30.0 g; Folic acid, 4.0 g; Biotin, 0.20 g; Vit C, 100.00 g.

**Table 5:** Growth performance of broiler birds as influenced by different treatments, single cell protein level and protein source during different phases

Protein source	SCP (%)	Tr. No.	Body weight gain (g/bird)				Feed intake (g/bird)				Feed conversion ratio (g feed/g gain)				Livability percent
		
			Starter	Grower	Finisher	Total	Starter	Grower	Finisher	Total	Starter	Grower	Finisher	Total	
Soybean meal	0	T_1_	323.7^bc^	260.0^a^	1,471.9^abc^	2,062.5^bc^	467.9^ab^	456.6^a^	2,701.9^ab^	3,626.4^ab^	1.446^cd^	1.758^cd^	1.838^d^^e^	1.766^e^	85.0
	2.5	T_2_	331.1^c^	295.1^b^	1,548.9^c^	2,172.0^cd^	473.3^ab^	504.1^bc^	2,791.1^abc^	3,768.5^abc^	1.430^c^	1.708^ab^	1.814^d^	1.740^d^	95.0
	5	T_3_	356.0^d^	298.9^bc^	1,540.1^c^	2,194.6^d^	504.9^c^	504.9^bc^	2,800.5^abc^	3,810.2^abc^	1.418^bc^	1.690^ab^	1.783^c^	1.712^c^	92.5
	7.5	T_4_	309.8^ab^	248.6^a^	1,428.5^ab^	1,987.9^ab^	458.4^a^	446.5^a^	2,666.2^ab^	3,571.0^a^	1.482^e^	1.795^d^^e^	1.867^f^	1.798^f^	97.5
	10	T_5_	302.4^a^	243.9^a^	1,385.1^a^	1,931.4^a^	471.8^ab^	449.9^a^	2,641.9^a^	3,563.6^a^	1.559^f^	1.847^f^	1.903^g^	1.842^g^	100.0
Fish meal	0	T_6_	326.6^bc^	311.1^bcd^	1,668.2^d^	2,310.6^e^	453.1^a^	513.6^c^	2,981.2^c^	3,947.8^c^	1.387^ab^	1.704^ab^	1.772^c^	1.709^c^	92.5
	2.5	T_7_	356.6^d^	321.4^cd^	1,672.5^d^	2,351.4^e^	489.0^bc^	542.8^c^	2,891.0^bc^	3,922.8^c^	1.371^a^	1.689^a^	1.726^b^	1.667^b^	97.5
	5	T_8_	359.2^d^	323.8^d^	1,681.6^d^	2,364.6^e^	487.8^bc^	538.5^c^	2,836.6^abc^	3,862.8^bc^	1.358^a^	1.664^a^	1.688^a^	1.635^a^	97.5
	7.5	T_9_	319.2^abc^	301.8^bcd^	1,510.5^bc^	2,131.1^cd^	470.8^ab^	523.6^c^	2,770.4^abc^	3,764.8^abc^	1.476^d^^e^	1.736^bc^	1.850^ef^	1.777^e^	97.5
	10	T_10_	312.1^ab^	260.0^a^	1,387.2^a^	1,964.0^ab^	477.1^abc^	467.1^ab^	2,620.5^a^	3,564.7^a^	1.529^f^	1.830^ef^	1.901^g^	1.832^g^	100.0
p-value^1)^			<0.001	<0.001	<0.001	<0.001	0.013	<0.001	0.037	0.003	<0.001	0.001	0.001	<0.001	0.132
Effect of SCP level															
	0		325.1^b^	285.6^b^	1,574.3^c^	2,191.9^c^	460.5^a^	485.1^a^	2,841.5^b^	3,787.1^bc^	1.417^b^	1.731^b^	1.805^c^	1.737^c^	88.8^a^
	2.5		343.8^c^	308.2^c^	1,612.3^c^	2,264.1^cd^	481.1^bc^	523.4^b^	2,841.1^b^	3,845.6^c^	1.401^ab^	1.799^a^	1.770^b^	1.704^b^	96.3^b^
	5		357.6^d^	311.3^c^	1,614.7^c^	2,284.2^d^	496.3^c^	521.7^b^	2,818.5^b^	3,836.5^c^	1.388^a^	1.677^a^	1.736^a^	1.673^a^	95.0^ab^
	7.5		314.5^ab^	275.2^b^	1,469.5^b^	2,059.9^b^	464.6^ab^	485.1^a^	2,718.3^ab^	3,667.9^ab^	1.479^c^	1.766^c^	1.859^d^	1.788^d^	97.5^b^
	10		307.3^a^	252.0^a^	1,386.1^a^	1,947.1^a^	474.4^ab^	458.5^a^	2,631.2^a^	3,564.2^a^	1.544^d^	1.839^d^	1.902^e^	1.837^e^	100.0^b^
p-value^1)^			<0.001	<0.001	<0.001	<0.001	0.003	<0.001	0.030	0.004	<0.001	<0.001	<0.001	<0.001	0.037
Effect of protein source															
Soybean meal			324.6	269.3	1,472.3	2,065.3	475.3	472.4	2,720.3	3,668.0	1.467	1.760	1.841	1.772	94.0
Fish meal			334.7	303.6	1,584.7	2,225.1	475.5	517.1	2,819.9	3,812.6	1.424	1.725	1.788	1.724	97.0
p-value^1)^			0.006	<0.001	<0.001	<0.001	0.960	<0.001	0.043	0.006	<0.001	0.001	<0.001	<0.001	0.183
SCP level and protein source interaction															
p-value^1)^			0.277	0.062	0.082	0.089	0.231	0.243	0.356	0.286	0.101	0.502	<0.001	<0.001	0.779
SEM			2.58	3.51	15.94	17.45	3.98	6.05	33.40	34.52	0.01	0.01	0.00	0.00	1.56

SCP, single cell protein; SEM, standard error of the mean.

1) An effect with a probability of less than 0.05 is considered significant.

a–d Means with in the same column bearing different superscripts differ significantly.

**Table 6:** Carcass characteristics and organometry of experimental broiler birds as influenced by different treatments, single cell protein level and protein source

Protein source	SCP %	Tr. No.	Dressing percent	Breast	Wings	Thigh	Abd Fat	Meat:Bone ratio	Liver	Heart	Gizzard	Bursa	Spleen	Thymus
Soybean meal	0	T_1_	74.49	24.42^ab^	7.75^b^	9.55^ab^	2.69	3.21^a^	1.853^bc^	0.545^c^	1.938^bcd^	0.068^abcd^	0.090^ab^	0.250^ab^
	2.5	T_2_	75.74	27.36^e^	6.80^a^	9.16^ab^	2.74	3.43^ab^	1.860^bc^	0.430^ab^	1.898^abcd^	0.093^e^	0.100^abc^	0.240^ab^
	5	T_3_	75.92	26.96^cd^	7.92^b^	9.73^bc^	2.87	3.75^abc^	1.963^bcd^	0.430^ab^	1.758^abc^	0.053^ab^	0.100^abc^	0.230^a^
	7.5	T_4_	74.53	23.63^a^	7.81^b^	9.93^bc^	2.79	3.89^bcd^	2.073^bcd^	0.458^ab^	1.785^abc^	0.060^abc^	0.150^e^	0.238^ab^
	10	T_5_	75.33	24.10^a^	8.38^b^	10.33^cd^	2.96	3.87^bcd^	2.148^cd^	0.455^ab^	1.618^a^	0.073^bcd^^e^	0.115^bcd^	0.265^b^
Fish meal	0	T_6_	75.97	25.88^cd^	7.72^b^	9.97^bc^	3.08	4.95^g^	1.430^a^	0.433^ab^	1.640^ab^	0.050^a^	0.085^a^	0.230^a^
	2.5	T_7_	75.88	27.24^d^^e^	7.94^b^	8.98^a^	2.81	4.57^ef^	1.768^b^	0.410^a^	2.000^cd^	0.058^abc^	0.125^cd^^e^	0.233^a^
	5	T_8_	77.08	25.62^bc^	7.68^b^	9.90^bc^	2.85	4.37^d^^e^	2.058^bcd^	0.435^ab^	2.100^cd^	0.065^abcd^	0.138^d^^e^	0.243^ab^
	7.5	T_9_	75.51	24.73^abc^	8.20^b^	11.02^e^	2.86	4.21^cd^^e^	1.998^bcd^	0.513^c^	1.890^abcd^	0.085^d^^e^	0.130^d^^e^	0.258^ab^
	10	T_10_	75.87	24.20^a^	8.45^b^	10.46^cd^	2.95	3.30^bcd^	2.220^d^	0.463^b^	1.626^ab^	0.074^cd^^e^	0.120^cd^	0.266^b^
p-value^1)^			0.756	<0.001	0.007	<0.001	0.611	<0.001	<0.001	<0.001	0.005	<0.001	<0.001	0.037
Effect of SCP level														
	0		75.23	25.15^b^	7.74^ab^	9.76^b^	2.88	4.08	1.641^a^	0.489^d^	1.789^ab^	0.059^a^	0.088^a^	0.240^a^
	2.5		75.81	27.30^c^	7.37^a^	9.26^a^	2.77	4.00	1.814^a^	0.420^a^	1.949^b^	0.075^b^	0.113^b^	0.236^a^
	5		76.50	25.79^b^	7.80^ab^	9.80^b^	2.86	4.16	2.010^b^	0.433^ab^	1.929^b^	0.059^a^	0.119^b^	0.236^a^
	7.5		75.02	24.18^a^	8.00^bc^	10.48^c^	2.80	4.05	2.035^b^	0.485^d^	1.838^b^	0.073^b^	0.140^c^	0.248^ab^
	10		75.60	24.15^a^	8.41^c^	10.40^c^	3.00	3.85	2.184^b^	0.459^bc^	1.622^a^	0.073^b^	0.118^b^	0.266^b^
p-value^1)^			0.558	<0.001	0.007	<0.001	0.705	0.74	<0.001	<0.001	0.009	0.02	<0.001	0.013
Effect of protein source														
Soybean meal			75.20	25.09	7.73	9.82	2.80	3.63	1.979	0.464	1.799	0.069	0.110	0.245
Fish meal			76.06	25.53	8.00	10.06	2.90	4.38	1.895	0.451	1.851	0.066	0.120	0.246
p-value^1)^			0.149	0.136	0.13	0.11	0.217	<0.001	0.163	0.181	0.398	0.508	0.129	0.835
SCP level and protein source interaction														
p-value^1)^			0.958	0.225	0.119	0.019	0.476	<0.001	0.04	<0.001	0.031	<0.001	0.012	0.242
SEM			0.42	0.21	0.12	0.11	2.74	0.08		0.01	0.04	0.00	0.004	0.004

SCP, single cell protein; SEM, standard error of the mean.

1) An effect with a probability of less than 0.05 is considered significant.

a–d Means with in the same column bearing different superscripts differ significantly.

**Table 7:** Length, microbial count and pH of small intestine of experimental broiler birds as influenced by different treatments, single cell protein level and protein source

Protein source	SCP %	Tr. No.	Intestinal length (cm/100 g body weight)			Microbial-count (cfu/g)								Intestine pH

						Duodenum		Ileum		Jejunum		Ileo-caecal		
				
			Duodenum	Ileum	Jejunum	10 μL (count×10^3^)	100 μL (count×10^2^)	10 μL (count×10^3^)	100 μL (count×10^2^)	10 μL (count×10^3^)	100 μL (count×10^2^)	10 μL (count×10^3^)	100 μL (count×10^2^)	
Soybean meal	0	T_1_	1.17^ab^	3.55^ab^	3.52^ab^	111.3	347.3	107.1	350.9	115.6	367.3	117.9	351.6	6.6
	2.5	T_2_	1.21^ab^	3.65^ab^	3.49^ab^	110.0	344.6	105.6	345.9	119.0	378.0	111.9	333.6	6.6
	5	T_3_	1.10^a^	3.40^a^	3.39^ab^	108.5	342.6	110.0	360.5	116.5	369.9	116.1	346.4	6.5
	7.5	T_4_	1.21^ab^	3.59^ab^	3.59^ab^	110.3	360.8	107.6	352.4	116.8	370.9	113.6	338.9	6.6
	10	T_5_	1.28^b^	3.79^ab^	3.76^b^	108.0	362.5	110.1	360.8	117.5	373.3	110.3	328.8	6.8
Fish meal	0	T_6_	1.09^a^	3.32^a^	3.29^a^	106.8	362.8	109.9	359.9	117.0	371.8	111.9	333.6	6.5
	2.5	T_7_	1.15^ab^	3.20^a^	3.23^a^	111.3	356.9	111.4	364.9	120.0	381.1	113.4	338.1	6.9
	5	T_8_	1.15^ab^	3.36^a^	3.39^ab^	107.8	377.0	113.3	371.0	119.5	379.5	112.0	334.0	6.5
	7.5	T_9_	1.23^ab^	3.59^b^	3.64^ab^	107.5	372.5	111.9	366.6	120.4	382.3	115.9	345.6	6.8
	10	T_10_	1.27^b^	3.80^b^	3.77^b^	109.0	361.0	112.4	361.8	121.1	384.8	111.3	331.8	6.7
p-value^1)^			0.097	0.01	0.041	0.935	0.303	0.899	0.912	0.996	0.996	0.872	0.872	0.102
Effect of SCP level														
	0		1.13^a^	3.43^a^	3.40^a^	109.0	355.0	108.5	355.4	116.3	369.5	114.9	342.6	6.6^ab^
	2.5		1.18^ab^	3.38^a^	3.36^a^	110.6	350.8	108.5	355.4	119.5	379.6	112.6	335.9	6.8^b^
	5		1.12^a^	3.38^a^	3.39^a^	108.1	359.8	111.6	365.8	118.0	374.7	114.1	340.2	6.5^a^
	7.5		1.22^ab^	3.61^ab^	3.61^ab^	108.9	366.6	109.8	359.5	118.6	376.6	114.8	342.3	6.7^ab^
	10		1.28^b^	3.76^b^	3.76^b^	108.5	361.8	111.3	361.3	119.3	379.0	110.8	330.3	6.8^b^
p-value^1)^			0.016	0.003	0.008	0.887	0.590	0.858	0.889	0.956	0.956	0.742	0.742	0.048
Effect of protein source														
Soybean meal			1.19	3.57	3.55	109.1	351.6	108.1	354.1	117.1	371.9	114.0	339.9	6.6
Fish meal			1.18	3.45	3.46	108.5	366.0	111.8	364.8	119.6	379.9	112.9	336.6	6.7
p-value^1)^			0.65	0.111	0.287	0.476	0.030	0.118	0.149	0.375	0.375	0.629	0.629	0.366
SCP level and protein source interaction														
p-value^1)^			0.686	0.467	0.621	0.756	0.540	0.990	0.956	0.997	0.997	0.688	0.688	0.355
SEM			0.02	0.08	0.06	1.14	4.61	1.63	5.20	2.00	6.35	1.57	7.43	0.05

SCP, single cell protein; SEM, standard error of the mean.

1) An effect with a probability of less than 0.05 is considered significant.

a,b Means with in the same column bearing different superscripts differ significantly.

**Table 8:** Intestinal morphometry of experimental broiler birds as influenced by different treatments, single cell protein level and protein source

Protein source	SCP %	Tr. No.	Duodenum			Jejunum			Ileum		
		
			Villi length (μm)	Crypt depth (μm)	Villi:crypt ratio	Villi length (μm)	Crypt depth (μm)	Villi:crypt ratio	Villi length (μm)	Crypt depth (μm)	Villi:crypt ratio
Soybean meal	0	T_1_	2,375.8^b^	268.8^a^	9.1	1,206.2^cd^	155.6^b^	7.8	843.6	137.2	6.2
	2.5	T_2_	2,171.6^ab^	227.6^a^	9.7	1,203.8^cd^	153.8^b^	7.9	850.0	133.0	6.4
	5	T_3_	2,777.8^c^	257.8^a^	10.8	1,287.2^d^	156.6^b^	8.3	869.4	134.4	6.5
	7.5	T_4_	2,044.2^a^	225.8^a^	9.2	1,161.8^bcd^	151.4^ab^	7.7	798.4	131.2	6.1
	10	T_5_	2,031.6^a^	228.8^a^	8.9	1,015.6^a^	135.0^a^	7.7	768.6	130.6	5.9
Fish meal	0	T_6_	2,035.8^a^	223.2^a^	9.2	1,155.0^bc^	146.6^ab^	7.9	838.4	133.6	6.3
	2.5	T_7_	2,205.4^ab^	236.8^a^	9.3	1,165.6^bcd^	145.6^ab^	8.0	821.6	130.2	6.3
	5	T_8_	3,106.6^d^	319.2^b^	9.8	1,178.2^bcd^	144.0^ab^	8.2	828.4	124.2	6.7
	7.5	T_9_	2,145.6^ab^	240.2^a^	9.1	1,061.8^ab^	138.4^ab^	7.7	770.8	127.6	6.0
	10	T_10_	2,031.6^a^	228.8^a^	8.9	1,019.8^a^	133.4^a^	7.7	768.6	130.6	5.9
p-value^1)^			<0.001	0.001	0.194	<0.001	0.047	0.992	0.195	0.628	0.688
Effect of SCP level											
	0		2,205.8^b^	246.0^a^	9.1^a^	1,180.6^bc^	151.1^b^	7.9	841.0^b^	135.4	6.2
	2.5		2,188.5^ab^	232.2^a^	9.5^ab^	1,184.7^bc^	149.7^b^	8.0	835.8^b^	131.6	6.4
	5		2,942.2^c^	288.5^b^	10.3^b^	1,232.7^c^	150.3^b^	8.2	848.9^b^	129.3	6.6
	7.5		2,094.9^ab^	233.0^a^	9.1^a^	1,111.8^b^	144.9^ab^	7.7	784.6^ab^	129.4	6.1
	10		2,031.6^a^	228.8^a^	8.9^a^	1,017.7^a^	134.2^a^	7.7	768.6^a^	130.6	5.9
p-value^1)^			<0.001	0.001	0.047	<0.001	0.029	0.778	0.036	0.567	0.208
Effect of protein source											
Soybean meal			2,280.2	241.8	9.5	1,174.9	150.5	7.9	826.0	133.3	6.2
Fish meal			2,305.0	249.6	9.3	1,116.1	141.6	7.9	805.6	129.2	6.3
p-value^1)^			0.61	0.399	0.411	0.026	0.019	0.833	0.297	0.127	0.851
SCP level and protein source interaction											
p-value^1)^			0.002	0.017	0.757	0.617	0.863	1	0.958	0.794	0.992
SEM			34.08	6.54	0.21	17.98	2.56	0.21	13.67	1.83	0.14

SCP, single cell protein; SEM, standard error of the mean.

1) An effect with a probability of less than 0.05 is considered significant.

a–d Means in the same column bearing different superscripts differ significant.
